# Reinforcement Learning-Based Satellite Attitude Stabilization Method for Non-Cooperative Target Capturing

**DOI:** 10.3390/s18124331

**Published:** 2018-12-07

**Authors:** Zhong Ma, Yuejiao Wang, Yidai Yang, Zhuping Wang, Lei Tang, Stephen Ackland

**Affiliations:** 1 Xi’an Microelectronics Technology Institute, Xi’an 710065, China; mazhong@mail.com (Z.M.); y88y01d07@sina.com (Y.Y.); zxjwl@126.com (Z.W.); tanglei@163.com (L.T.); 2 Centre for Computational Intelligence, De Montfort University, Gateway House, Leicester LE1 9BH, UK; smackland@dmu.ac.uk

**Keywords:** deep reinforcement learning, satellite attitude control, dynamic environment, Deep Q Network, parametric uncertainty

## Abstract

When a satellite performs complex tasks such as discarding a payload or capturing a non-cooperative target, it will encounter sudden changes in the attitude and mass parameters, causing unstable flying and rolling of the satellite. In such circumstances, the change of the movement and mass characteristics are unpredictable. Thus, the traditional attitude control methods are unable to stabilize the satellite since they are dependent on the mass parameters of the controlled object. In this paper, we proposed a reinforcement learning method to re-stabilize the attitude of a satellite under such circumstances. Specifically, we discretize the continuous control torque, and build a neural network model that can output the discretized control torque to control the satellite. A dynamics simulation environment of the satellite is built, and the deep Q Network algorithm is then performed to train the neural network in this simulation environment. The reward of the training is the stabilization of the satellite. Simulation experiments illustrate that, with the iteration of training progresses, the neural network model gradually learned to re-stabilize the attitude of a satellite after unknown disturbance. As a contrast, the traditional PD (Proportion Differential) controller was unable to re-stabilize the satellite due to its dependence on the mass parameters. The proposed method adopts self-learning to control satellite attitudes, shows considerable intelligence and certain universality, and has a strong application potential for future intelligent control of satellites performing complex space tasks.

## 1. Introduction

Good attitude control methods are crucial for the stable on-orbit operation of satellites. During the on-orbit operation of the satellite, the motion state and mass characteristics of the system will change. Such changes include long-term consumption of fuel, changes in the configuration of the spacecraft (such as the expansion of the solar panels or some large antennas), the capture and release of the payloads on-orbit (such as the release of the satellite from a spaceship, the capture of the target, the removing of orbital garbage, etc.), and the rendezvous and docking with other spacecrafts. Many of these changes are violent (such as capture, release of the satellite, docking with the target, etc.) and unpredictable (such as the operation of non-cooperative targets, the removal of orbital waste, etc.) [1].

Most existing attitude control algorithms rely on the mass parameters of the controlled object (including mass, moment of inertia, etc.), and the mass parameters need to be identified by various means [2]. In this case, it is difficult to provide accurate parameter identification [3]. The dynamic model of this kind of system is complex and has strong nonlinearity, which may easily lead to the failure of the existing common attitude control systems [4]. Therefore, there is an urgent need for a highly autonomous attitude control technology with a considerable intelligence to solve the high-performance control problem of spacecraft under the on-orbit change of the mass characteristics of the spacecraft that is difficult to control by traditional methods.

Aiming at the attitude control in such complex situations, Yoon H et al. proposed a novel control law for a nonlinear Hamiltonian MIMO (Multiple-Input Multiple-Output) system, aiming at the existence of inertial uncertainty in spacecraft attitude control [5]. Queiroz M S D et al. designed the nonlinear adaptive control method using the dynamic model of the complete system and proved that the global stability of tracking error of the closed-loop system converges when the interference is unknown [6]. Miao et al. used an adaptive sliding mode control strategy to solve the vibration problem in the process of maneuvering large flexible spacecraft [7]. However, the current attitude control algorithms for spacecraft are lacking in modern computational intelligence tools as they are usually designed for specific applications that do not have universal applicability. Therefore, with the continuous increase in the complexity of space exploration missions, it is necessary to design an attitude control method with considerable intelligence.

At present, the actual problems in satellite attitude control are external interference problems, uncertainties in moment of inertia, and nonlinear problems of models [8]. Backstepping attitude control is very effective for nonlinear system control, and the operation steps are simple; it can solve the problem of difficult construction of the system Lyapunov function [9]. A nonlinear control approach for satellite attitude stabilization maneuver is presented in [10]. The controller is developed by using the backstepping control technique. External disturbances and actuator constraints are all considered during the simulation. Simulation results revealed the control validity of the proposed controller. The paper [11] designs an adaptive prediction backstepping controller (APBC) under the consideration of estimation error of the moment of inertia, flexible vibration and disturbance torques. Compared with the traditional PD (Proportion Differential) controller, the APBC has good control performance on eliminating the effect of time delay and provides estimations of moment of inertia parameters online.

Deep reinforcement is a technique that directly learns control strategies from high-dimensional raw data [12]. In order to solve the computer’s control problem from perception to decision-making, and thus to realize universal artificial intelligence, it has been rapidly developed in the past two years and has made break-throughs in the fields of video games, Go, and robotics [13]. Deep Q Networks (DQNs) are one such deep reinforcement learning algorithm that combines neural networks and Q-Learning, where the input is the original image data, and the output is the value evaluation (Q value) corresponding to each action [14]. In 2013, DeepMind proposed a DQN algorithm [15] to train artificial intelligence (AI) to play games on the Atari platform. The model surpassed the performance of previous algorithms on 6 of the 7 games tested, with 3 of them exceeding the human level, showing the great potential of such algorithms in intelligent decision-making.

Aiming at the problem of space satellite intelligent attitude control, this paper proposes a deep reinforcement learning algorithm for autonomous controlling. The algorithm overcomes the limitation of the existing method that relies on mass parameters and solves the problem of satellite attitude instability encountered with sudden random disturbance.

The main research contributions of this work are as follows: (1) We proposed an intelligent attitude control method, based on the deep reinforcement learning technology. This method brings the artificial intelligence into the satellite control domain. The merit of the proposed method is twofold. First, the method does not rely on the mass parameters of the satellite, and thus can solve the problem of sudden changes in the attitude and mass parameters encountered by the satellite when performing complex tasks, such as capturing an unknown target. Second, instead of being designed by experts, the control law is learned by the algorithm itself. It can be easily adapted to solve other satellite control problems or incorporated with other sensors, such as cameras, to further improve the control performance. (2) We built a dynamic simulation environment to simulate the dynamic of the satellite while it is under unknown disturbances in its attitude and mass parameters. The simulation environment can be used to train the artificial intelligence algorithm within it, and to validate the existing control methods.

The remaining sections are organized as follows. In the next section, a satellite intelligent attitude control method subject to parametric uncertainty has been designed. The method is composed of two parts: the construction of the dynamic environment and the building and training of the deep reinforcement model. Numerical simulation and results analysis are demonstrated in Section 3. Finally, Section 4 concludes this paper.

## 2. Satellite Attitude Control

The satellite intelligent attitude control problem addressed in this paper is defined formally as follows:

*Given a satellite that maintains a stable attitude angular velocity on the orbital coordinate system, how can we re-stabilize the satellite attitude back to the initial state after it has encountered an unknown disturbance both in attitude and mass parameters?*

To simulate such a process, we build a simulation environment in which a random disturbance torque is applied to the satellite based on the dynamic model, and the moment of inertia is randomly changed to simulate changes in satellite mass parameters at the same time. The proposed method based on deep reinforcement learning should continuously output control torque in this state to control the satellite to restore a stable flight attitude.

**Figure 1:**
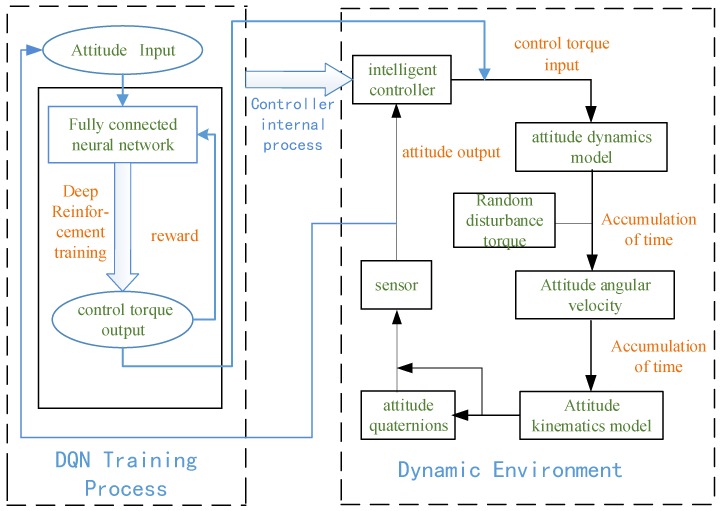
Sketch map of satellite attitude control method based on deep reinforcement learning.

The traditional satellite control method such as PD controllers struggle to re-stabilize the satellite after it encounters unknown perturbation because of their strict reliance on the mass parameters. In this paper, we use deep reinforcement learning to solve this problem. The process can be divided into two steps: first, to construct a dynamic environment, which models the dynamics of the spacecraft flying in near-earth space. The inputs of this model are the control torques, and the outputs of the model are the attitude angular velocities of the spacecraft. The second is to use a DQN algorithm to perform deep reinforcement training of the control torque. The design process is shown in Figure 1. The sensor is fixedly connected to the simulation environment and provides the intelligent controller with the required measurement input information such as attitude angular velocity and attitude quaternions.

The proposed satellite attitude stabilization method is a learning-based method, with the learning phase performed on the ground. After the learning phase, the neural network model is obtained, and this model can be used to stabilize the satellite on board. Concretely, we have established a fully connected neural network as the agent. The agent takes the attitude angular velocity and attitude quaternion of the satellite as input and the control torque of the satellite as the output. Then the agent is trained within the satellite’s dynamic simulation environment, the parameters of the neural network model are continuously updated in the training phase. After the training, we get a trained agent and this can be applied to the satellite. The agent will act as the brain to control the flight attitude of the satellite during the flight. In this paper, the effectiveness of the trained agent is validated in the dynamic simulation environment.

**Figure 2:**
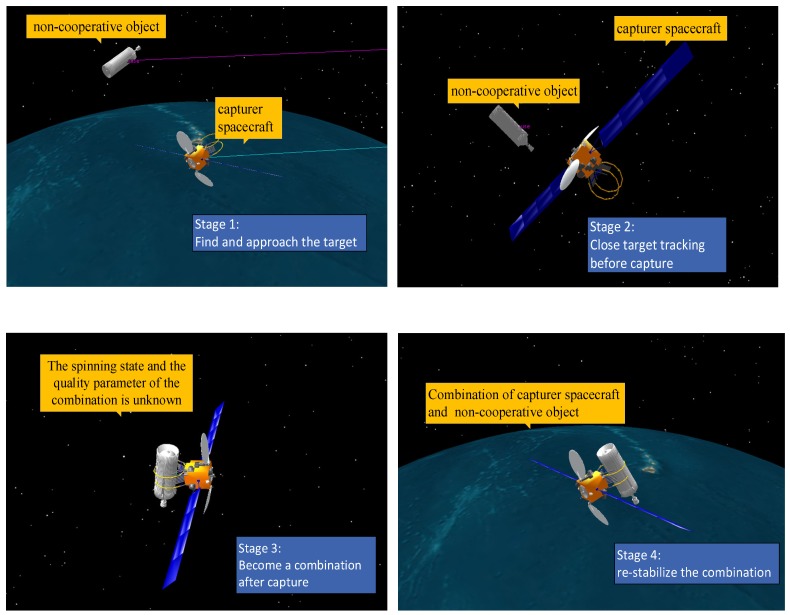
Example of a spacecraft capturing a non-cooperative object.

Figure 2 shows the visualization results of a typical process from our simulation environment, where the capturer spacecraft is capturing a non-cooperative object in space. The object usually has an unknown flight state, possibly spinning with unknown angular velocity, and unknown mass parameters. After the capturer spacecraft has successfully captured the object, the combined objects have unknown angular velocity, and the mass parameters, such as the centroid of the combination, will become unknown as well. The proposed method tries to re-stabilize the combination in such situations.

### 2.1. Construction of Dynamic Environment

In order to study the problem of satellite attitude control, the dynamic of the satellite in space is built based on the orbital coordinate system; that is, the origin of the coordinates is at the centroid of the satellite, the Z axis points to the earth center, the Y axis is along the negative normal line of the orbital plane of the satellite, with the X, Y and Z axes constituting a right-handed system. Simultaneously, in order to describe the motion of a satellite attitude under the action of external torques, correct attitude dynamics and kinematics models must be established [16].

(1) Establishing differential equations for attitude dynamics to solve the attitude angular velocity of the satellite.

The dynamic model of the satellite can be described by the Euler dynamic equation of a single rigid body as follows:

(1) $$\dot{\omega }=I^{-1}\left(T-\omega \times \left(I\cdot \omega \right)\right),$$

where $T={\left[\begin{array}{ccc} T_{x} & T_{y} & T_{z} \end{array}\right]}^{T}$ is the control torque acting on the centroid of the rigid body, I∈R^{3×3} is the moment of inertia matrix of the rigid body, which reflects the mass parameters of a spacecraft, and I is a symmetric matrix with a positive and largest diagonal elements. $\omega ={\left[\begin{array}{ccc} \omega_{x} & \omega_{y} & \omega_{z} \end{array}\right]}^{T}$ is the attitude angular velocity of the rigid body. $\dot{\omega }$ is the first derivative of ω. If the initial value of the attitude angular velocity is known as $\omega_{0}$, given control torque T, and setting I to a fixed value, it is possible to obtain the attitude angular velocity of the satellite at any time by solving the upper formula.

(2) Establishing differential equations for attitude kinematics to solve the attitude quaternion of the satellite.

According to Euler’s finite rotation theorem, any angular displacement of a rigid body around a fixed point can be obtained by turning an angle around an axis passing through the point. Thus, the attitude parameter between two coordinate systems can be described by a unit vector e in the reference coordinate system and the angle Φ around this axis. The unit vector e has three components in this coordinate system, and these four parameters form the Eulerian axis/angle parameters $\left(\Phi ,e_{x},e_{y},e_{z}\right)$.

The quaternion can be obtained as $Q={\left[\begin{array}{cccc} q_{0} & q_{1} & q_{2} & q_{3} \end{array}\right]}^{T}$ by transforming the Euler axis/angle parameter concept. The relationship between the two is as follows:

$$q_{0}=\cos\frac{\Phi }{2},q_{1}=e_{x}\sin\frac{\Phi }{2},q_{0}=e_{y}\sin\frac{\Phi }{2},q_{0}=e_{z}\sin\frac{\Phi }{2}$$

As with other attitude parameters, quaternions have three independent parameters. The fourth parameter is non-independent and satisfies constraints $q_{0}^{2}+q_{1}^{2}+q_{2}^{2}+q_{3}^{2}=1$.

Because the attitude of the satellite can be described by the attitude quaternion, the quaternion is used to characterize the change of the attitude of the satellite that the human eyes can observe. The following equation is the quaternion-based attitude kinematics equation of the satellite, $\omega ={\left[\begin{array}{ccc} \omega_{x} & \omega_{y} & \omega_{z} \end{array}\right]}^{T}$ is the attitude angular velocity of the satellite. $q={\left[\begin{array}{cccc} q_{0} & q_{1} & q_{2} & q_{3} \end{array}\right]}^{T}$ is the quaternion of the satellite, $\dot{Q}={\left[\begin{array}{cccc} {\dot{q}}_{0} & {\dot{q}}_{1} & {\dot{q}}_{2} & {\dot{q}}_{3} \end{array}\right]}^{T}$ is the first derivative of $Q={\left[\begin{array}{cccc} q_{0} & q_{1} & q_{2} & q_{3} \end{array}\right]}^{T}$. If the satellite is known at its initial attitude quaternion $Q_{0}$, the attitude of the satellite can be represented at any time by integrating.

(2) $$\left[\begin{array}{c} {\dot{q}}_{0}\\ {\dot{q}}_{1}\\ {\dot{q}}_{2}\\ {\dot{q}}_{3} \end{array}\right]=\frac{1}{2}\left[\begin{array}{cccc} q_{0} & -q_{1} & -q_{2} & -q_{3}\\ q_{1} & q_{0} & -q_{3} & q_{2}\\ q_{2} & q_{3} & q_{0} & -q_{1}\\ q_{3} & -q_{2} & q_{1} & q_{0} \end{array}\right]\left[\begin{array}{c} 0\\ \omega_{x}\\ \omega_{y}\\ \omega_{z} \end{array}\right],$$

(3) Constructing dynamic environment.

According to the spacecraft dynamics model, the dynamic environment in which the control torque and satellite attitude feedback to each other is constructed as follows. In particular, to simulate normal disturbances such as gravity, radio, and electromagnetic radiation in space, we add constant disturbance torque to the dynamics model which is described by random function.

Step 1: Randomly initialize the attitude angular velocity $\omega_{0}$, attitude quaternion $Q_{0}$ of the satellite;

Step 2: Given a random disturbance torque TS;

Step 3: Equations (1) and (2) are integrated sequentially to solve the attitude angular velocity $\omega_{i}$ and attitude quaternion $Q_{i}$;

The attitude angular velocity and attitude quaternion are used as the input of the neural network for subsequent deep reinforcement learning training.

### 2.2. Construction of DQN Training Process

Intelligent attitude control of the satellite is a complex, high-dimensional issue. The neural network is regarded as the control agent which decides how much control torque should be given based on the current motion state of the satellite. As mentioned previously, a two-layer fully connected neural network is used as the calculation component, the inputs of this network are the satellite’s current attitude angular velocity and attitude quaternion, the outputs of this network are the probabilities of each possible actions (e.g., a certain control torque). The actual action is chosen from all these actions according to the probabilities. The probabilistic strategy of sampling actions will encourage the action that leads to good results and suppress the action that leads to bad results. In summary, the deep reinforcement training process based on DQN is as the following Algorithm 1:

**Algorithm 1.** Control torque training process based on Deep Q Network (DQN) algorithm1. Initialize the capacity of the experience pool D for N, which is used to store training samples; 2. Use the deep neural network as the Q-value network to initialize weight parameters θ; 3. Set the total number of control task training as M, loop start: Initialize the network input state $x_{1}$, and calculate network output $a_{1}$. 1) Randomly select the action $a_{t}$ with probability epsilon (decreasing with the number of iterations) or the maximum Q value $\underset{a}{\arg\max}Q\left(x_{t},a;\theta \right)$ output through the network; 2) After performing $a_{t}$ in the environment, get reward $r_{t}$ and input $x_{t+1}$ for the next network; 3) Save the parameter vector $\left(x_{t},a_{t},r_{t},x_{t+1}\right)$ as D at the moment (D holds the state of N moments); 4) When D accumulates to a certain degree, the minibatch states are randomly taken out of D after each execution of 1–3 steps; 5) Calculate the target value for each state $\left(x_{j},a_{j},r_{j},x_{j+1}\right)$:
$$y_{j}=\{\begin{array}{c} r_{j},x_{j+1}\;terminates\;the\;task\\ r_{j}+\gamma \underset{a\prime }{\max}Q\left(x_{j+1},a\prime ;\theta \right),x_{j+1}\;does\;not\;terminate\;the\;task \end{array}$$ When the terminal in the randomly selected minibatch $\left(x_{j},a_{j},r_{j},x_{j+1}\right)$ is true, that is the deviation between the actual attitude angular velocity and the desired attitude angular velocity of the satellite falls into a predefined range. Then we think $x_{j+1}$ terminates, vice versa. γ is discount factor; 6) The network weight parameter is updated through SGD and the loss function is defined using the mean squared error ${\left(y_{j}-Q\left(x_{j},a_{j};\theta \right)\right)}^{2}$. Loop execution of the above 1–6 steps, continuous training model. 4. Multiple training to get the model.

### 2.3. Establishment of Deep Reinforcement Training

**Figure 3:**
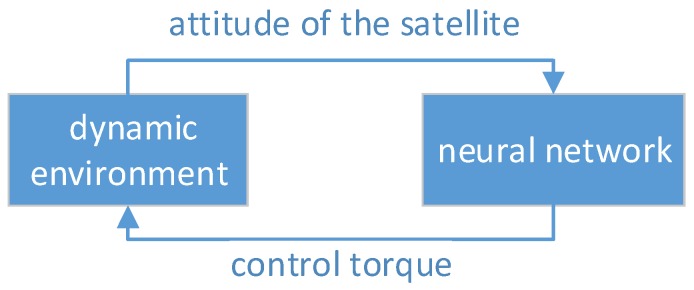
The interactive process of the dynamic environment and the neural network.

In this section, we use the deep reinforcement learning algorithm—Deep Q Network, to perform the intelligent autonomous attitude control training for the satellite in the dynamic environment described in the previous section. We built a fully connected neural network as the intelligent agent, which takes the attitude of the satellite as input, and outputs the control torque for the satellite. Unlike a convolutional neural network, each neuron is connected to only a small number of neurons [17], and the fully connected neural network is connected to all neurons in the upper layer. The input layer of the fully connected neural network has 7 nodes, corresponding to the 7-dimensional representation of the satellite attitude. The hidden layer has two layers; the first layer has 1024 nodes, and the second layer has 2048 nodes. The output layer has 7 nodes, corresponding to the seven types of value vectors after the control torque is discretized. Weight matrix and offset are corresponding values and the weight parameters of the fully connected neural network are obtained by using the DQN algorithm. As shown in Figure 3, at each time step, the control torque is sent back into the dynamic environment, and the dynamic environment continues to output the attitude of the satellite to feed the neural network for continuous deep reinforcement training.

By continuous self-learning and self-evolution, the weight value of the policy network is constantly updated. It is mainly divided into the following steps:

(1) Discretization of Control Torque

DQN is a discrete control-oriented algorithm; that is, the output of the action is discrete. DQN cannot handle continuous action, because the update of the Q value needs to be achieved by seeking the largest action. However, in the problem of spacecraft controlling, the control torque is continuous and high-dimensional and cannot be solved directly using the DQN method. Therefore, the control torque’s output is discretized here.

The control torque of the spacecraft is assumed to be a three-dimensional vector $T\in R^{3}$, with a possible value range $1.0e^{-2}\times \left\{-1,0,1\right\}$ of each direction component in $T={\left[T_{x},T_{y},T_{z}\right]}^{T}$, that is only one direction component of control torque has only one certain value in the set of $1.0e^{-2}\times \left\{-1,0,1\right\}$ for each maneuver, and the other components have a value of zero. There are 7 kinds of torque distribution methods according to the above range of values, with the flag vector for each of which set as [1,0,0,0,0,0,0], [0,1,0,0,0,0,0], [0,0,1,0,0,0,0], [0,0,0,1,0,0,0], [0,0,0,0,1,0,0], [0,0,0,0,0,1,0], [0,0,0,0,0,0,1], respectively. These vectors can represent the control torque for an iterative update of the Q value. Among them, [1,0,0,0,0,0,0] represents that the control torque of the agent output is zero, that is, the spacecraft has no external torque applied, and only relies on its original angular velocity to continue to rotate. The correspondence between the control torque and flag vector is shown in the following Table 1.

Actually, one kind of attitude control is discontinuous, i.e., jet propulsion. Thus, discretization of control torque is reasonable. The problem in satellite control is called phase plane. One example of practical application with 7 × 2 × 3 = 42 control modes is widely used in the field of attitude control for both large and small spacecrafts where phase plane control laws are applied. However, the number 42 is just the result of permutations and combinations for all the jet propulsion engines equipped on spacecrafts. In fact, each of the 7 kinds of control modes proposed in this paper could be the orthonormal basement of any discontinuous attitude control law for 3-aixs rigid body, including phase plane and others as long as the control step-size is small enough.

(2) Design of Reward Function and Terminating Condition

The goal of deep reinforcement training is to output the optimal control torque of the satellite, so that the deviation between the initial attitude angular velocity $\omega_{0}$ and the satellite attitude angular velocity $\omega_{i}$ obtained through the dynamic model is almost zero. However, the goal of the training is to get as much reward as possible. Therefore, the reward function needs to have the nature of a decreasing function, where the smaller the angular velocity difference (defined as $\omega_{i}$ − $\omega_{0}$) is, the greater the reward. In this paper, we use the Gaussian function to construct the reward function:

(3) $$g\left(\omega_{i}-\omega_{0}\right)=\frac{1}{\sqrt{2\pi }}e^{-\frac{1}{2}{\left(\omega_{i}-\omega_{0}\right)}^{2}},$$

We have also set the terminating condition of the training process of the DQN algorithm. The criteria of whether to complete the training is dependent on whether the torque can control the satellite to restore to a stable attitude. Here, we calculate the deviation between the actual attitude angular velocity and the desired attitude angular velocity of the satellite at each iteration, when the deviation falls into a predefined range, the training process is terminated. The predefined range is the deviation of the satellite attitude angular velocity in the three-directional components being less than 1.0e−06.

## 3. Simulation Experiment and Results Analysis

To verify the effectiveness of the proposed method, simulation experiments were performed. Firstly, the dynamic model based on constant disturbance is used to simulate the movement of satellites in space. Then, to simulate the tasks such as target capture or payload release performed by satellites, an instantaneous burst random disturbance torque is applied to the satellite based on the above dynamic model, and the moment of inertia has randomly been changed to simulate changes in satellite mass parameters at the same time. The proposed method subject to parametric uncertainty should continuously output control torque in this state to control the satellite to restore a stable flight attitude. In this paper, I∈R^{3×3} is the moment of inertia matrix of the satellite, which reflects the mass parameters of the satellite, and I is a symmetric matrix with a positive and largest diagonal elements. To simulate changes in satellite mass parameters, I is randomly been changed by adding a lower order 3 × 3 random symmetric matrix based on a basic matrix $\left[\begin{array}{ccc} 2.0257 & 0.6498 & 1.1226\\ 0.6498 & 0.7998 & 0.1833\\ 1.1226 & 0.1833 & 1.2753 \end{array}\right]$, each element in the random symmetric matrix is drawing from an uniform distribution with range of [0,1].

Before the start of each simulation, the satellite initial attitude quaternion and angular velocities are initialized randomly, and the entire satellite attitude maneuvering process is observed based on sensor acquisition information. When the attitude quaternion and angular velocity deviation no longer decrease with time, the attitude maneuvering process is considered to be finished, and the attitude accuracy and stability of the attitude control system are evaluated based on the final angular velocity deviation.

First, we define the mass of the satellite to be m = 5 kg, randomly initialize the attitude angular velocity $\omega_{0}$, attitude quaternion $Q_{0}$ and the moment of inertia matrix I of the satellite. Then, to simulate ubiquitous disturbances such as gravity, radiation, and pressure of the sunlight, etc., we add ubiquitous random disturbance torque to the dynamics model of the satellite, this disturbance is described by random function $TS=1.0e^{-3}r\times {\left[-1,0,1\right]}^{T}$, where r is a random variable subject to a uniform distribution, which ranges [0,1]. Specifically, an instantaneous burst random disturbance torque which simulates the tasks such as target capture performed by satellites is defined as $TR=1.0e^{-2}\times {\left[r1,r2,r3\right]}^{T}$, where r1,r2,r3 are randomly generated numbers. The expected experiment results should be, after this disturbance, the attitude control algorithm continuously outputs the control torque, so that the satellite’s attitude angular velocity can converge to a certain value, and the deviation between this value and the desired attitude angular velocity tends to zero, indicating that the attitude control algorithm is effective. For comparison, this paper also tests the traditional attitude control method based on PD controller. The algorithm is implemented using the Anaconda3 software package and TensorFlow deep learning software framework.

### 3.1. Attitude Control Experiment Based on DQN Training

The definition of the number of iterations is 3000, the capacity of the experience pool is 500, and the discount factor is 0.99. The initial value of the neural network weight parameter is 0.01. The dynamic model based on constant disturbance is taken as the environment, and the moment of inertia matrix is randomly selected to perform deep reinforcement training based on the DQN algorithm.

To verify that the intelligent algorithm based on deep reinforcement learning can achieve attitude stabilization efficiently when performing spatial non-cooperative target capturing, the algorithm has been tested several times in the simulation environment. 

**Figure 4:**
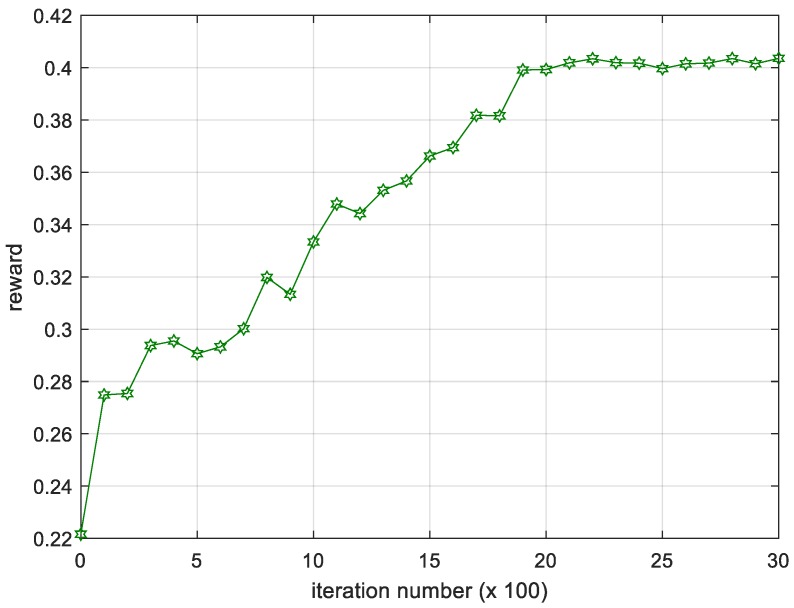
Iteration process of the average reward.

We calculate the average results of the reward value per 100 iterations in different experiments and show the changing trend of reward. As shown in the Figure 4, we can see that from the 500th iteration, the average reward is increasing rapidly. When the 2000th iteration is reached, the reward value has stabilized and has only small fluctuations. The fluctuations are mainly caused by constant disturbance. After entering the stationary period, there is not much improvement, which shows that the training is converged after about 2000 iterations.

The attitude angular velocity and its error were calculated and recorded per 100 iterations, and the training result of the 3000 iterations is shown in the figure below.

**Figure 5:**
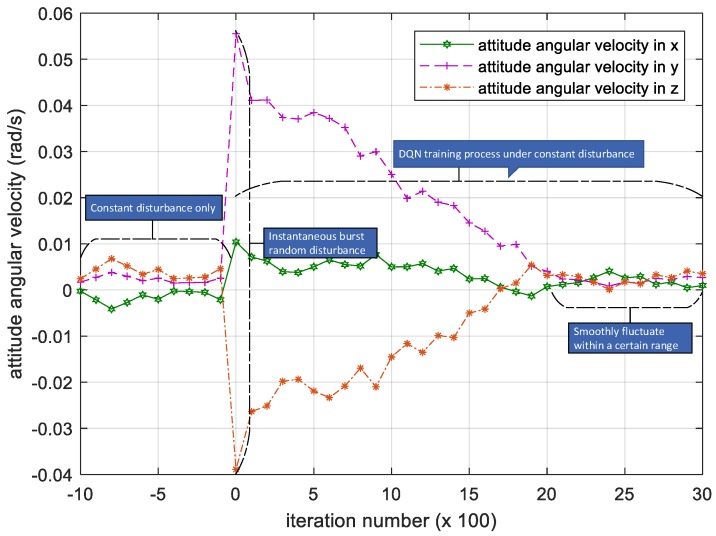
Iteration process of the attitude angular velocity. Please note, the iteration number in this figure reflects the time span of the experiment.

As seen in Figure 5, the period in which the iteration number is between −10 and 0 is when DQN algorithm has not been applied. At that time, the satellite is running smoothly under constant disturbance, so the attitude angular velocity is showing a random change. After the instantaneous burst random disturbance torque is loaded in the satellite, the attitude angular velocity is increasing instantaneously. Then the deep reinforcement algorithm DQN starts the process of training torque control of the satellite.

As the number of iterations increases, the attenuation change of attitude angular velocity in the three-directional components indicates that the satellite’s attitude of the three-directional components all decrease and converge, indicating that the satellite attitude reaches a steady state, and the DQN-based attitude control method subject to parametric uncertainty is effective.

If there is no constant disturbance, the attitude angular velocity of the satellite after DQN training will return to the initial stable value after the convergence is stabilized, that is, at the 2000th iteration. However, due to the existence of constant disturbance, the attitude angular velocity will still fluctuate within a certain range, and the range of amplitude variation will be reduced by a little before the occurrence of the instantaneous disturbance under the action of DQN.

**Figure 6:**
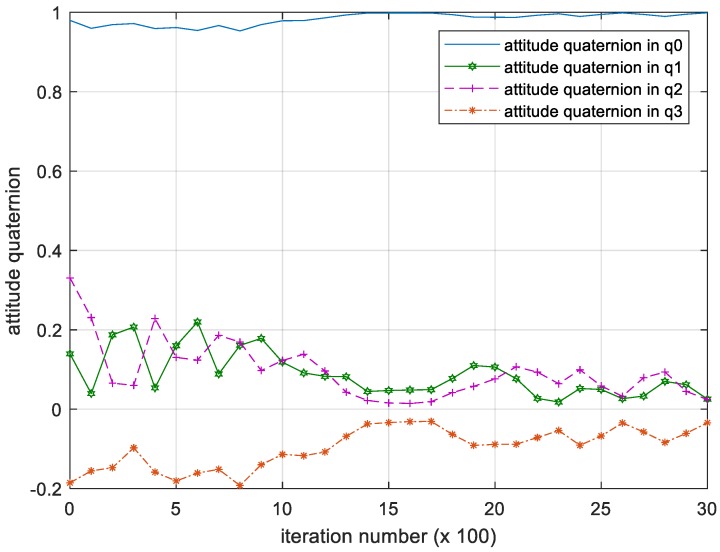
Iteration process of the attitude quaternion. Please note, the iteration number in this figure reflects the time span of the experiment.

**Figure 7:**
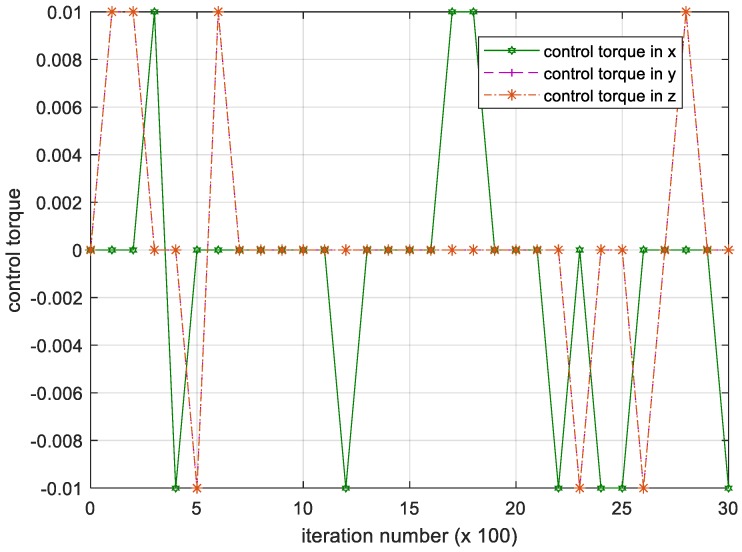
Iteration process of the control torque. Please note, the iteration number in this figure reflects the time span of the experiment.

We have shown the iterative process of the attitude angular velocity in Figure 5. The numerical results of time histories of state variables such as attitude quaternion and control torque are shown in Figure 6 and Figure 7 below.

**Figure 8:**
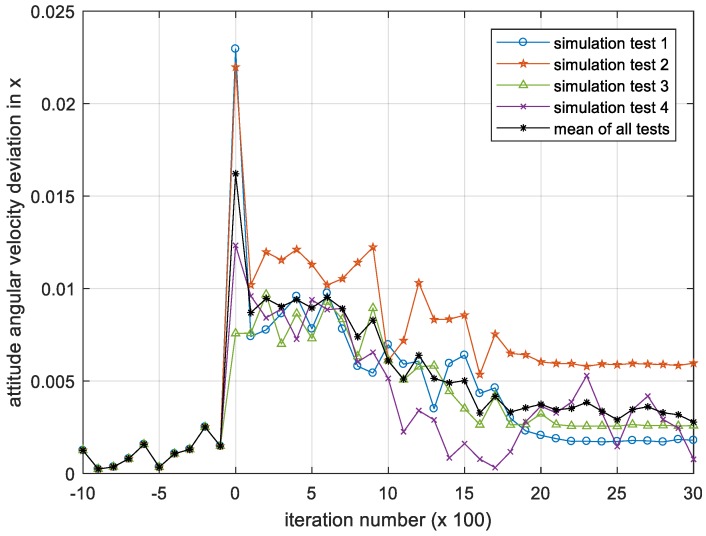
Iteration process of the attitude angular velocity deviation in x. Please note, the iteration number in this figure reflects the time span of the experiment.

**Figure 9:**
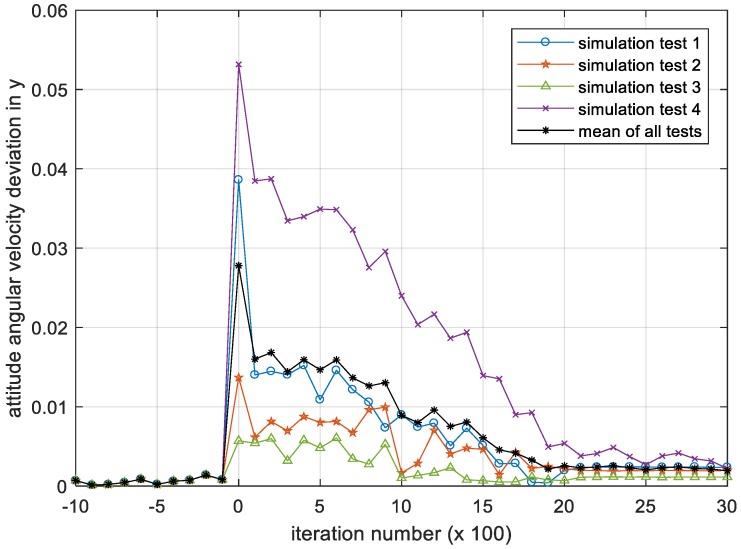
Iteration process of the attitude angular velocity deviation in y. Please note, the iteration number in this figure reflects the time span of the experiment.

**Figure 10:**
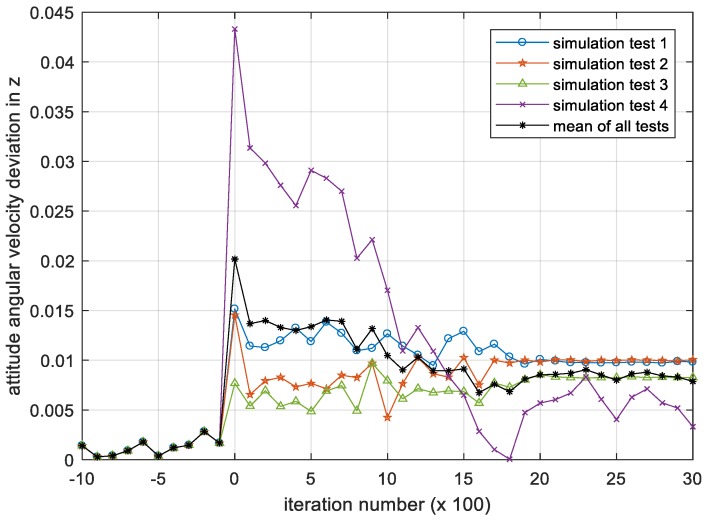
Iteration process of the attitude angular velocity deviation in z. Please note, the iteration number in this figure reflects the time span of the experiment.

Figure 8, Figure 9 and Figure 10 show the iterative process of the attitude angular velocity deviation in the three-directional components under four simulation experiments. It can be seen from all tests that as the number of iterations increases, the mean value of the deviation in the x, y and z axes all shows a downward trend and gradually converges to a stationary state, indicating that the DQN-based attitude control method has the performance advantage of stabilizing the attitude of the satellite in the case that the parameters are difficult to identify.

### 3.2. Attitude Control Experiment Based on PD Controller

The PD controller strictly depends on the mass parameter, i.e. the moment of inertia I of the controlled object. In the figures below, the attitude angular velocity and its deviation of the satellite over 3000 iterations is shown when I takes a random disturbance. 

**Figure 11:**
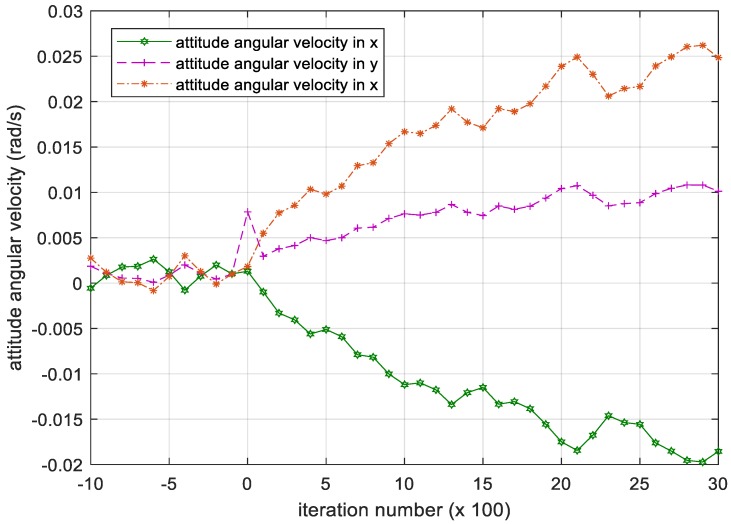
Iteration process of the attitude angular velocity in PD controller. Please note, the iteration number in this figure reflects the time span of the experiment.

**Figure 12:**
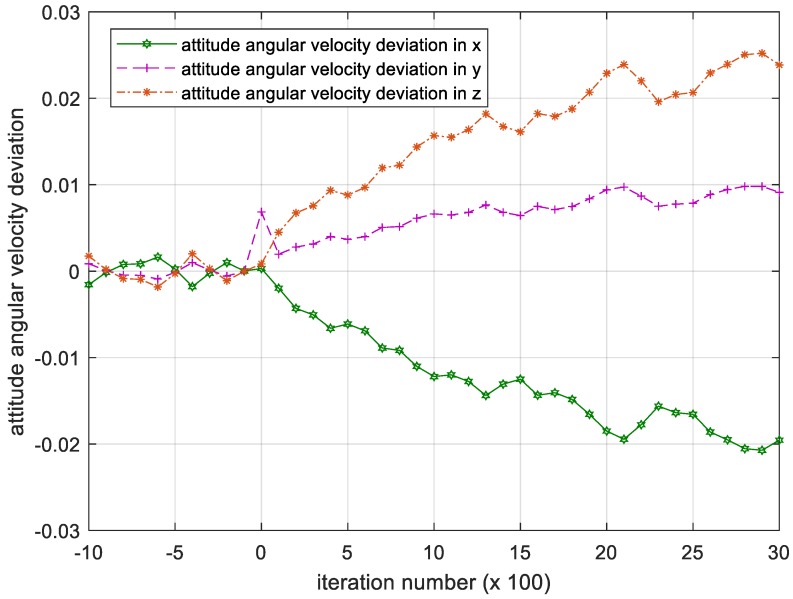
Iteration process of the attitude angular velocity deviation in PD controller. Please note, the iteration number in this figure reflects the time span of the experiment.

Figure 11 demonstrates that the attitude angular velocity gradually increases as the number of iterations increases, failing to converge. This divergent deviation between the attitude angular velocity and the desired attitude angular velocity shown in Figure 12 indicates that the satellite cannot maintain the attitude stabilization.

Considering the uncertainties of the mass parameters such as the moment of inertia matrix, the comparison with Section 3.1 also verifies that deep reinforcement learning technology still has the advantage of stabilizing satellite attitude under random parameter changes.

It can be seen that the optimal intelligent output of the control torque is obtained as the attitude angular velocity tends to be stable as a reward. The space-enhanced satellite attitude control-based deep reinforcement learning algorithm has been able to autonomously stabilize satellite attitude after sudden random disturbance and is superior to conventional PD controllers in maintaining satellite attitude stabilization.

### 3.3. Attitude Control Experiment Based on Backstepping Controller

**Figure 13:**
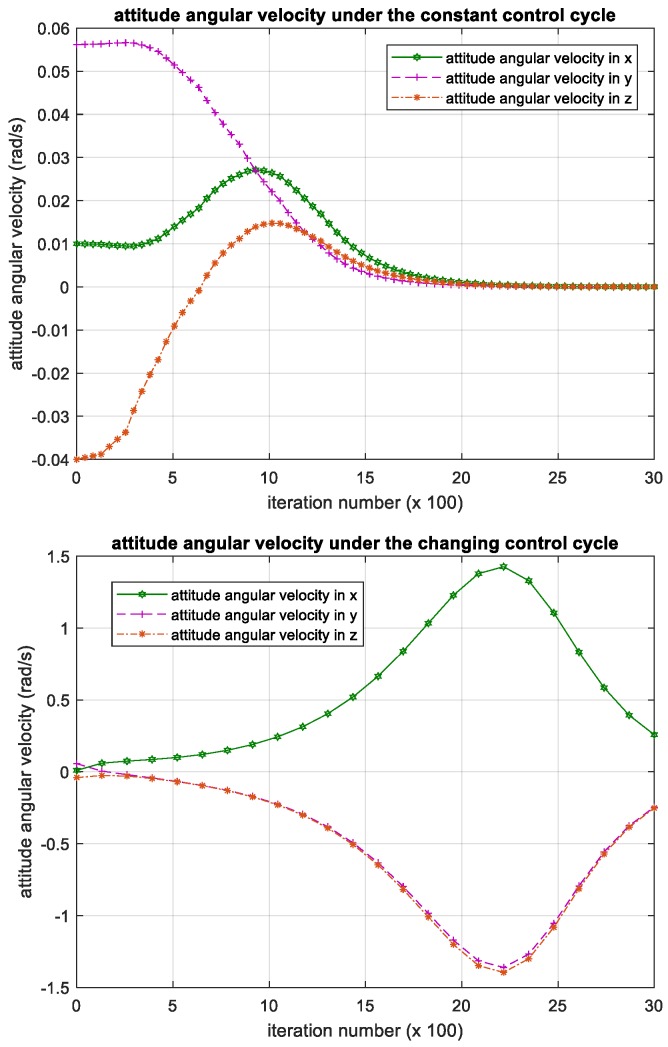
Iteration process of the attitude angular velocity in backstepping controller. Please note, the iteration number in this figure reflects the time span of the experiment.

**Figure 14:**
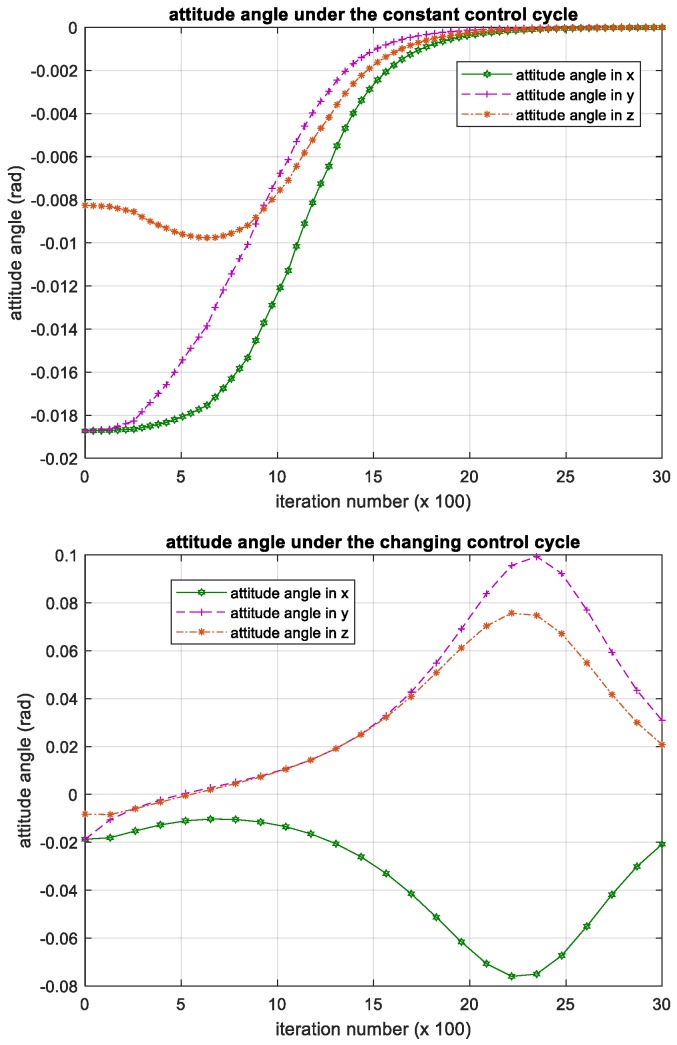
Iteration process of the attitude angle in backstepping controller. Please note, the iteration number in this figure reflects the time span of the experiment.

**Figure 15:**
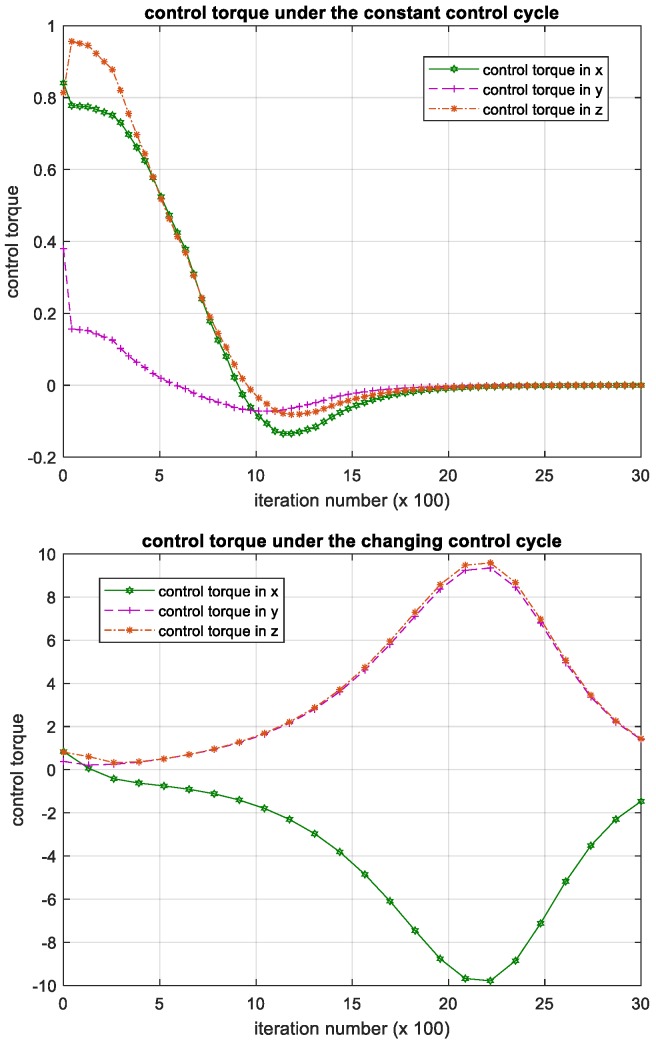
Iteration process of the control torque in backstepping controller. Please note, the iteration number in this figure reflects the time span of the experiment.

To further validate the effectiveness of proposed method, we compared the proposed method with a kind of robust attitude control method based on backstepping. This method uses the adaptive control theory to stabilize the satellites, and the effectiveness of this method can be proved analytically with the Lyapunov method. The experiment results of this method for the same problem are shown in Figure 13, Figure 14 and Figure 15.

The results show that the backstepping controller has robustness against mass parameter uncertainty. However, the backstepping controller can only handle the constant control cycle; when the control cycle is constant, iteration process of the attitude angular velocity, attitude angle and control torque can converge. When the control cycle changes during the iteration process, it becomes divergent. Compared with the backstepping controller, our method based on deep reinforcement learning has competitive performance, while it allows the control cycle changes during iteration. That is, regardless of whether the control cycle changes or not, our method can control the satellite to restore a stable flight attitude.

## 4. Conclusions

Aiming at the problem of sudden changes in satellite attitude encountered when performing complex tasks, this paper uses deep reinforcement learning technology to restore the attitude of the satellite. A dynamic model of mutual feedback of control torque and attitude angular velocity was established, setting the attitude dynamics environment for deep reinforcement learning training. The attitude control problem is applied to the updating of the Q value in the DQN algorithm by discretizing the control torque output. The optimal intelligent control torque output is obtained by taking the attitude angular velocity as stability. Simulation results illustrate that the proposed method can restore the satellite after its motion and mass state were changed to an unknown state, as contrasted with the traditional PD controller, which failed to solve this problem. The proposed deep reinforcement learning method is an adaptive control method that solves the problem of satellite attitude control, Furthermore, it paves a way for many other complex space tasks, such as non-cooperative target capturing, and maneuvering near a small celestial body whose gravity field is unknown, etc. For future work, we plan to train and validate the method on a semi-physical simulation platform consisting of a three-dimensional turntable and an actuator under the complex space simulation environment.

## Figures and Tables

**Table 1:** Discretization of control torque.

Direction Component of Control Torque T	Flag Vector
$T_{x}=T_{y}=T_{z}=0$	[1,0,0,0,0,0,0]
$T_{x}=-1.0e-02,\;T_{y}=T_{z}=0$	[0,1,0,0,0,0,0]
$T_{x}=1.0e-02,\;T_{y}=T_{z}=0$	[0,0,1,0,0,0,0]
$T_{x}=0,\;T_{y}=-1.0e-02,\;T_{z}=0$	[0,0,0,1,0,0,0]
$T_{x}=0,\;T_{y}=1.0e-02,\;T_{z}=0$	[0,0,0,0,1,0,0]
$T_{x}=T_{y}=0,\;T_{z}=-1.0e-02$	[0,0,0,0,0,1,0]
$T_{x}=T_{y}=0,\;T_{z}=1.0e-02$	[0,0,0,0,0,0,1]
